# Innominate vs. Axillary Artery Cannulation in Aortic Surgery: a Systematic Review and Meta-Analysis

**DOI:** 10.21470/1678-9741-2018-0272

**Published:** 2019

**Authors:** Amer Harky, Jeffrey SK Chan, Christiana Bithas, Alexander Hof, Monira Sharif, Saied Froghi, Mohamad Bashir

**Affiliations:** 1 Department of Vascular Surgery, Countess of Chester, Chester, United Kingdom of Great Britain and Northern Ireland.; 2 Faculty of Medicine, The Chinese University of Hong Kong, Hong Kong, Region of the People's Republic of China.; 3 Department of Cardiovascular Surgery, Heinrich-Heine-University, Medical Faculty, Dusseldrof, Germany.; 4 School of Medicine, University of Liverpool, Liverpool, United Kingdom of Great Britain and Northern Ireland.; 5 Department of Surgery, Imperial College NHS Trust, Hammersmith Hospital, Du Cane Rd London, United Kingdom of Great Britain and Northern Ireland.; 6 Manchester Royal Infirmary, United Kingdom of Great Britain and Northern Ireland.

**Keywords:** Dissecting Aneurysm - Surgery, Thoracic Surgical Procedures, Axillary Artery, Peripheral Catherization - Methods, Treatment Outcomes

## Abstract

**Objective:**

To investigate whether axillary artery cannulation has supremacy over innominate artery cannulation in thoracic aortic surgery.

**Methods:**

A comprehensive search was undertaken among the four major databases (PubMed, Excerpta Medica dataBASE [EMBASE], Scopus, and Ovid) to identify all randomized and nonrandomized controlled trials comparing axillary to innominate artery cannulation in thoracic aortic surgery. Databases were evaluated and assessed up to March 2017.

**Results:**

Only three studies fulfilled the criteria for this meta-analysis, including 534 patients. Cardiopulmonary bypass time was significantly shorter in the innominate group (*P*=0.004). However, the innominate group had significantly higher risk of prolonged intubation > 48 hours (*P*=0.04) than the axillary group. Further analysis revealed no significant difference between the innominate and axillary groups for deep hypothermic circulatory arrest time (*P*=0.06). The relative risks for temporary and permanent neurological deficits as well as in-hospital mortality were not significantly different for both groups (*P*=0.90, *P*=0.49, and *P*=0.55, respectively). Length of hospital stay was similar for both groups.

**Conclusion:**

There is no superiority of axillary over innominate artery cannulation in thoracic aortic surgery in terms of perioperative outcomes; however, as the studies were limited, larger scale comparative studies are required to provide a solid evidence base for choosing optimal arterial cannulation site.

**Table:** 

Abbreviations, acronyms & symbols				
AATS	= American Association for Thoracic Surgery		NYHA	= New York Heart Association
ACP	= Antegrade cerebral perfusion		PND	= Permanent neurological deficits
Art	= Artery		POD	= Presentation on demand
BMI	= Body mass index		PRISMA	= Preferred Reporting Items for Systematic Reviews and Meta-Analyses
DHCA	= Deep hypothermic circulatory arrest		RAA	= Right axillary artery
CI	= Confidence interval		RCP	= Retrograde cerebral perfusion
CKD	= Chronic kidney disease		SD	= Standard deviation
COPD	= Chronic obstructive pulmonary disease		TND	= Temporary neurological deficits
CPB	= Cardiopulmonary bypass		USA	= United States of America
EMBASE	= Excerpta Medica dataBASE			
IA	= Innominate artery			

## INTRODUCTION

Thoracic aortic surgery entails complex and major procedures performed in specialized centres by dedicated aortic surgeons. Such procedures are associated with significant morbidity and mortality rates. Therefore, optimizing perioperative outcomes in these circumstances is crucial for a satisfactory perioperative recovery^[1]^. A key factor in determining such outcomes is the site of the arterial cannulation, which, upon surgical planning, should be tailored as per individual patient^[2]^. Di Eusanio et al.^[3]^, among others, have shown that selecting an ideal arterial cannulation site can have a favourable impact on reducing neurological complications and lower mortality rates in patient with acute type A aortic dissections. Regardless of cerebral perfusion and temperature management, neurological injuries are the highest risk complications during aortic arch surgery^[1]^. Currently, to minimize such complications during aortic arch surgery, deep hypothermic circulatory arrest (DHCA) with antegrade cerebral perfusion (ACP) is most widely used^[1]^. Aside from ACP, retrograde cerebral perfusion (RCP) is also feasible and established in conjunction with DHCA.

Over the past two decades, the ideal site of arterial in-flow has changed^[4-6]^. Initially, surgeons preferred to use femoral artery as the main arterial cannulation with RCP. However, more recently, central cannulation has become an alternative and safe perfusion site in acute aortic dissections or patients with severe aortic atherosclerosis^[7,8]^.

Our systematic review focuses on two main central cannulation sites: the right axillary artery (RAA) and the innominate artery (IA). Evidence from studies by Di Eusanio et al.^[3]^ suggests that cannulation of RAA in patients undergoing surgery for atherosclerotic aneurysm and organ malperfusion in acute type A aortic dissection can reduce the risk of cerebral embolism during the antegrade blood flow through the thoracoabdominal aorta. It is reported by Chu et al.^[9]^ that cannulating RAA for systematic perfusion during circulatory arrest results in half the incident rate of neurological events when compared to IA cannulation as an access site for providing cerebral protection during aortic surgery, although the difference was statistically insignificant.

Recently, the cannulation of IA has emerged as a popular choice over RAA cannulation^[10]^. It was first introduced by Banbury and Cosgrove for cannulation in proximal aortic surgery^[11]^. In recent studies by Chu et al.^[9]^ and Di Eusanio et al.^[3]^, IA cannulation demonstrated a valid and safe alternative to RAA cannulation, and it is also a simpler technique for establishing ACP as it requires no side graft in most cases. Furthermore, Garg et al.^[12]^ demonstrated that for aortic arch reconstruction, cannulation of IA for ACP and DHCA was both feasible and safe. Through adopting a technique to avoid separate axillary cut down incisions, IA cannulation reduces the overall time required for surgery, surgical mortality, and neurological injuries^[1,3,9]^. IA cannulation is technically simpler in comparison to RAA due to its larger size, providing an ideal blood flow rate during cardiopulmonary bypass (CPB)^[2]^. Furthermore, RAA involves increased risk of developing limb ischaemia, arm hyper-perfusion, and seroma^[1]^. Although evidence from Svensson et al.^[4]^ represents the global preference for IA cannulation over RAA through retrospective studies, there has not been any high quality data to prove that this technique is superior to RAA cannulation in providing appropriate cerebral protection.

This paper aims to establish an understanding of the ideal site of cannulation to enable the best perioperative outcome possible. Thus, we investigated whether axillary artery has supremacy over IA cannulation during thoracic aortic surgery.

## METHODS

### Literature Search Strategy and Inclusion Criteria

Electronic database searches and screening were performed by two reviewers independently using PubMed, Ovid, Scopus, and Excerpta Medica database (EMBASE) to identify all randomized and nonrandomized controlled trials up to March 2017 that compared axillary to IA cannulation in thoracic aortic surgery. Limits were placed to only include studies written in the English language that compared clinical outcomes such as perioperative outcomes and mortality rates. Non-comparative studies and studies that did not report clinical outcomes were excluded. The search terms included axillary, innominate, brachiocephalic, cannulation, aortic surgery, aorta, neurology, and outcomes. All search terms were combined with Boolean operators and searched as both key words and MeSH terms to ensure maximal sensitivity. After excluding articles based on title or abstract, the following full text articles that were selected had their reference lists searched for any potential further articles to be included in this review.

### Data Extraction and Critical Appraisal

The main outcome measures extracted included the following: in-hospital mortality, temporary and permanent neurological deficits, and length of stay. Other data were also extracted for assessment of perioperative characteristics of patients. The quality of the studies included was assessed by the Newcastle-Ottawa Scale, where each asterisk (*) represents one point, papers with seven or more points provide a quality study (Table 1).

**Table 1:** Newcastle-Ottawa Quality Assessment Scale.

Author	Selection				Comparability	Outcomes		
	Representation of patients with RAA cannulation	Selection of patients with IA cannulation	Ascertainment of exposure	Demonstration that the outcome of interest was not present at the start of study	Indication of surgery = *	Assessment of outcomes	Follow-up long enough for outcomes to occur	Adequacy of follow-up of cohorts
Svensson et al.^[4]^	*	*	*	*	*	*	*	*
Chu et al.^[9]^	*	*	*	*	*	*	*	*
Di Eusanio et al.^[3]^	*	*	*	*	*	*	*	*

IA=innominate artery; RAA=right axillary artery

### Statistical Analysis

Standard descriptive statistics (reported as means, with 95% of confidence interval [CI], were available) were used to summarize demographic and baseline data of the patients from all eligible studies. Meta-analysis of reported outcomes was performed on the reported in-hospital mortality, CPB time, DHCA time, and, separately, temporary and permanent neurological deficits.

Heterogeneity was predominantly reported as the Chi^2^ statistic, with Tau^2^ and I^2^ statistics also calculated. Random effect was estimated by the DerSimonian-Laird method. All statistical analyses were conducted with Review Manager Version 5.1.2 (Cochrane Collaboration, Software Update, Oxford, United Kingdom) and Stata Version 15.1 (StatCorp LLC, Texas, United States of America [USA]).

## RESULTS

### Study Characteristics

**Fig. 1:**
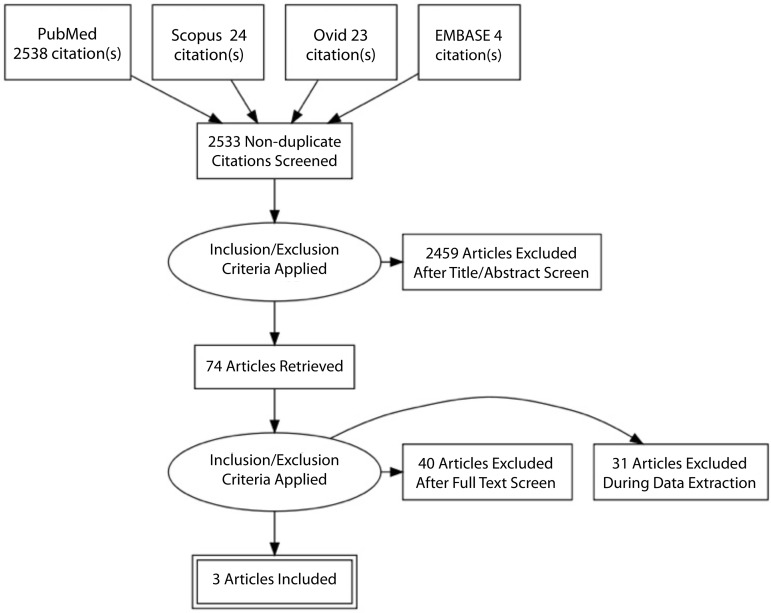
PRISMA chart of literature search. EMBASE=Excerpta Medica dataBASE; PRISMA=Preferred Reporting Items for Systematic Reviews and Meta-Analyses

The aforementioned strategy revealed 2589 articles, of which 74 were selected for full text review. During full text review, 62 papers were excluded as they were non-comparative studies and they have included only single site cannulation outcomes report. Furthermore, nine more papers were excluded as they were comparisons between central and peripheral cannulation, without giving the specific results of axillary *vs*. IA cannulation. Finally, three comparative observational studies were included in the study^[3,4,9]^. The search strategy performed is summarized by a Preferred Reporting Items for Systematic Reviews and Meta-Analyses (PRISMA) chart in Figure 1. A total of 534 patients were included in the analysis, of which 400 were cannulated via axillary artery and 134 were via IA. Characteristics of the included studies are summarized in Table 2.

**Table 2:** Study characteristics of the articles included in the systematic reviews and meta-analysis.

Author	Year	Country	Type	No. of patients	RAA (n)	IA (n)	Primary end points	Comments/Conclusion
Svensson et al.^[4]^	2004	USA	Prospective study	323	299	24	Stroke and mortality	Axillary inflow plus graft reduces stroke and is the method of choice for complex cardiac and cardio-aortic operations that necessitate circulatory arrest. Retrograde or antegrade perfusion is used selectively.
Chu et al.^[9]^	2016	Canada	Prospective study	140	74	66	Clinical and neurological outcomes	Axillary and IA cannulation for ACP during proximal aortic arch reconstructive surgery results in similarly excellent neurological outcomes. IA cannulation might reduce surgical time. Possible relevant differences in neurological and respiratory complications require assessment in randomized controlled trials.
Di Eusanio et al.^[3]^	2014	Italy	Prospective study	71	27	44	Perioperative comorbidities and in-hospital outcomes	RAA and IA were associated with similarly valid results. The choice between the two, based on the specific patient's characteristics, can improve outcomes after aortic surgery.

ACP=antegrade cerebral perfusion; IA=innominate artery; RAA=right axillary artery; USA=United States of America

### Perioperative Results

The key perioperative characteristics of patients included in this analysis are summarized in Table 3. Patients who had IA cannulation had higher body mass index (BMI) (28.4 ± 4.5 kg/m^2^ for IA cannulation *vs*. 27.25 ± 3.45 kg/m^2^ for RAA cannulation; *P*=0.0022) then those who had RAA cannulation, however IA patients had less rates of chronic obstructive pulmonary disease and history of smoking (21% for IA cannulation *vs*. 44% for RAA cannulation; *P*=0.03) than RAA patients. Moreover, RAA was cannulated in all cases of emergency thoracic aortic surgeries and was significantly more likely to be cannulated in non-emergency surgeries (84.40% for RAA cannulation *vs*. 15.60% for IA cannulation; *P*=0.002). There was no significant difference in total length of in-hospital stay among both cohorts of patients (*P*=0.121).

**Table 3:** Perioperative characteristics and postoperative outcomes of patients included in the analysis.

	Right axillary artery cannulation	Innominate artery cannulation	*P* value
No. of patients	N=400	N=134	-
Mean age (years) ±SD	63 ±12	62±11	0.3
Male (%)	278 (69.5)	61 (45.5)	-
NYHA III/IV (%)	17.75	5.22	-
Reoperation (%)	13 (3)	23 (17)	0.002
Hypertension (%)	268 (67)	60 (44)	0.01
Diabetes mellitus (%)	85 (21)	7 (5)	0.003
COPD (%)	227 (57)	32 (24)	<0.0001
BMI (mean ±SD)	27.25±3.45	28.4±4.5	0.0022
CKD/renal failure (%)	20 (5)	1 (1)	0.321
Smoking history - COPD (%)	176 (44)	28 (21)	0.03
Surgical acuity			
Emergency (%)	162 (40)	113 (85)	0.01
Non-emergency (%)	238 (60)	21 (15)	0.002
Operative data			
CPB (mins) (mean ±SD)	167.45±54.67	173.12±51.85	0.004
DHCA (mins) (mean ±SD)	29.14 ±23.55	38.48±31.32	0.06
Postoperative data			
TND^a^ (%)	12 (3)	13 (10)	0.90
PND^b^ (%)	14 (4)	6 (5)	0.49
Reoperation for bleeding (%)	25 (6)	11 (8)	0.93
In-hospital mortality (%)	24 (6)	7 (5)	0.55
Renal failure (%)	23 (6)	14 (10)	0.89
Cannulation related complications (%)	0.25	-	-
Prolonged intubation >48 hrs (%)	22 (6)	10 (8)	0.04
Sepsis (%)	32 (8)	1 (1)	0.95
Hospital stay - days (mean ± SD)	7±6	6±4	0.121

a Includes transient ischaemic attacks, delirium, and confusion.

b Includes stroke and hypoxic brain injury.

BMI=body-mass index; CKD=chronic kidney disease; COPD=chronic obstructive pulmonary disease; CPB=cardiopulmonary bypass; DHCA=deep hypothermic circulatory arrest; NYHA=New York Heart Association; PND=permanent neurological deficits; SD=standard deviation; TND=temporary neurological deficits

**Fig. 2:**
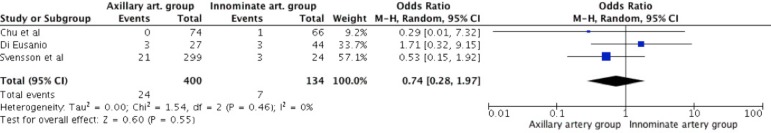
In-hospital mortality. Art=artery; CI=confidence interval

**Fig. 3:**
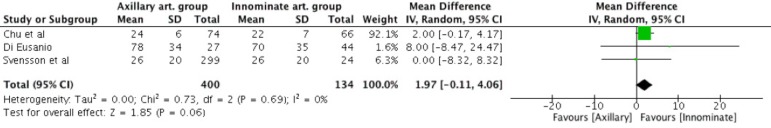
Deep hypothermic circulatory arrest time. Art.=artery; CI=confidence interval; SD=standard deviation

**Fig. 4:**
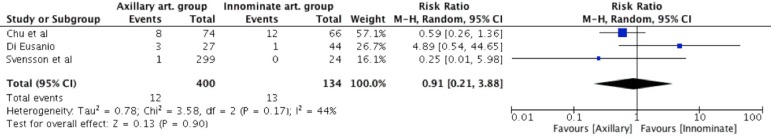
Temporary neurological deficit rate. Art.=artery; CI=confidence interval

**Fig. 5:**
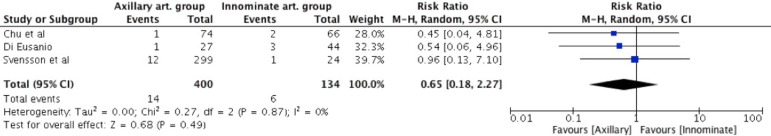
Permanent neurological deficit rate. Art.=artery; CI=confidence interval

**Fig. 6:**
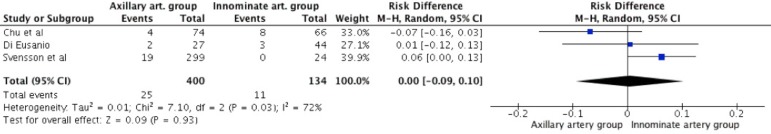
Rate of reoperation for bleeding. Art.=artery; CI=confidence interval

**Fig. 7:**
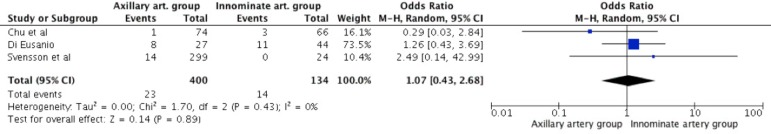
Rate of postoperative renal failure. Art.=artery; CI=confidence interval

**Fig. 8:**
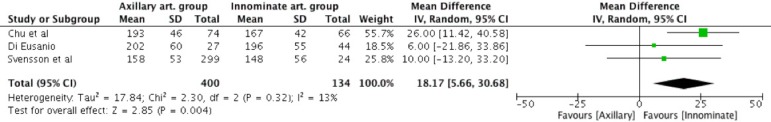
Cardiopulmonary bypass time. Art.=artery; CI=confidence interval; SD=standard deviation

**Fig. 9:**
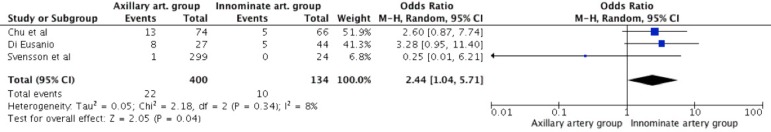
Rate of prolonged intubation >48 hours. Art.=artery; CI=confidence interval

Selected in-hospital postoperative outcomes were measured and they are summarized in Table 3. The meta-analysis was carried out for in-hospital mortality, DHCA time, CPB time, rates of temporary and permanent neurological deficits, rates of prolonged intubation > 48 hours, rates of reoperation for bleeding, and rates of postoperative renal failure. There was no significant difference between the two cannulations sites for in-hospital mortality rate (*P*=0.55; Figure 2), DHCA time (*P*=0.06; Figure 3), temporary (*P*=0.90; Figure 4) and permanent (*P*=0.49; Figure 5) neurological deficits, rates of reoperation for bleeding (*P*=0.93; Figure 6), and rates of postoperative renal failure (*P*=0.89; Figure 7). The data of the rates of reoperation for bleeding was significantly heterogenous (Chi^2^=7.10, df=2, *P*=0.03). On the other hand, CPB time was significantly shorter in cases where IA was cannulated (*P*=0.004; Figure 8) than in axillary cases. Nevertheless, IA cannulation was also associated with significantly higher risks of prolonged intubation > 48 hours (*P*=0.04; Figure 9) than axillary cannulation. Other measured in-hospital postoperative outcomes were not significantly different between the two groups, including the rate of sepsis and cannulation-related complications (*P*>0.05).

## DISCUSSION

In the past five decades, the optimal site for arterial cannulation has evolved according to attempts to improve perioperative outcomes such as survival rates and detrimental neurological impacts in patients who underwent complex aortic surgeries^[4,9,13]^. Examples of previously used cannulation sites include the femoral and subclavian arteries, and, more recently, the distal ascending aorta^[14]^. However, the distal ascending aortic inflow route of cannulation is associated with increased risks of lower limb ischaemia, lack of ACP use, dissection, and stroke^[4,13]^. As such, an overwhelming volume of studies have provided evidence of the beneficial outcomes of using cannulation sites amongst branches of the ascending aorta and the aortic arch, such as RAA and IA^[2,3,9]^. Cannulation of the axillary artery using side graft with ACP has been shown to provide better perioperative outcomes than classical cannulation sites^[9,13]^. More specifically, RAA cannulation reduces the probability of malperfusion, atheroma or calcified plaque disruption, and thromboembolic stroke^[4]^. More recently, IA is becoming the preferred site of cannulation during hemi-arch reconstruction. It provides similar advantages to those of RAA cannulation, such as lower rate of surgical complication, including neurological injury or surgical mortality^[9]^. On the other hand, IA is not risk free and it is also associated with risks, such as arterial dissection^[3,9]^.

With these trends in surgical approaches in mind, this meta-analysis aimed to compare the different intraoperative and postoperative outcomes of IA and RAA cannulations in patients undergoing thoracic aortic surgery. Pooling of the data from available evidence did not show any statistical difference (*P*=0.55) in the in-hospital mortality between RAA (6%) and IA (5.22%) cannulation. Both permanent and temporary neurological deficits have not been found to be significantly different between RAA and IA cannulation either; RAA and IA cannulation led to 3.5% and 4.48% cases of permanent neurological deficits (*P*=0.49), and 3% and 9.70% cases of temporary neurological deficits (*P*=0.90), respectively. This is consistent with previously reported findings, including the ones by Etz et al.^[8]^, which included 608 patients cannulated at the ascending aorta or RAA, finding no difference between RAA and other cannulations sites in terms of survival and neurological outcomes^[9]^.

Furthermore, there was no significant difference in post-operative sepsis incidence (*P*=0.95) for RAA (8%) compared to IA cannulation (0.75%), as well as post-operative renal failure (*P*=0.89), 5% and 0.75% for RAA and IA, respectively. Moreover, the analysis did not demonstrate any significant difference in the mean duration of DHCA in both groups of patients (29.14 ± 23.55 minutes for RAA cannulation and 38.48 ± 31.32 minutes for IA cannulation; *P*=0.06). These outcomes were consistent with the existing literature by Chu et al. (24 ± 5 minutes for RAA cannulation and 22 ± 7 minutes for IA cannulation respectively; *P*=0.001), with Di Eusanio et al. being also unable to demonstrate any significant difference (78 ± 34 minutes for RAA cannulation and 70 ± 35 minutes for IA cannulation*; P*=0.625)^[3,9]^.

In addition, this meta-analysis showed no significant difference in the rate of reoperation for bleeding (6.25% for RAA cannulation and 8.21% for IA cannulation; *P*=0.93). This confirms the findings from the study by Di Eusanio et al.^[3]^, who also found no significant difference in reoperation rate between the two approaches (48.1% for RAA cannulation and 52.3% for IA cannulation; *P*=0.463).

Nevertheless, IA cannulation was associated with shorter CPB duration (*P*=0.004), with a mean time of 173.12± 51.85 minutes and 167.45 ± 54.67 minutes for RAA and IA cannulation, respectively (*P*=0.004). This builds on the observed and statistically significant decrease in the CPB time of IA cannulation (202 ± 60 minutes and 196 ± 55 minutes for RAA and IA cannulation, respectively, *P*=0.727) by Di Eusanio et al.^[3]^, and it is reasonable in view of RAA cannulation as a more technically demanding and time-consuming procedure^[6,9]^. Though not specifically investigated in this meta-analysis for the setting of thoracic aortic surgery, CPB time has been shown to correlate independently with mortality and morbidity^[15,16]^. Moreover, shorter CPB time and technical demands in IA cannulation than in RAA cannulation may, in turn, reduce the mean procedural time and thus increase the efficiency and volume of care, especially in high-volume centres^[1,12]^. Therefore, our results provide a possible justification for the preference for IA over RAA cannulation.

On the other hand, IA cannulation has been found to be associated with higher rates of prolonged intubation > 48 hours (6% in RAA cannulation and 8% in IA cannulation; *P*=0.04). This happens despite statistically non-significant findings by Chu et al.^[9]^ (18% in RAA cannulation and 8% in IA cannulation; *P*=0.078) and Di Eusanio et al.^[3]^(30% in RAA cannulation and 11% in IA cannulation; *P*=0.055). This finding should be interpreted with caution since the data is heavily skewed by the outcomes reported in the study by Svensson et al.^[4]^ (1 out of 299 patients with RAA cannulation and 0 out of 24 patients with IA cannulation). Nonetheless, prolonged intubation has been shown to be associated with significantly higher mortality and respiratory complications^[17]^. As such, this may be a possible counterargument against the preferential choice of IA cannulation over RAA cannulation.

### Limitations

Interpretation of this meta-analysis must consider several limiting factors. Firstly, all the included studies are prospective studies without any randomization. This presents a significant source of potential bias which may confound the data. Secondly, not all variables were reported across all studies. Particularly, the length of stay in hospital and intensive care units were only reported by Chu et al.^[9]^, and, as such, it cannot be meta-analysed. This constitutes a significant limitation of this study, since the above two variables are important outcome measures, and the lack of them significantly impacts the comprehensiveness of the evaluation of RAA and IA cannulations. Thirdly, publication bias may have confounded the results, as observational studies with undesirable outcomes may not have published their results in full. Fourthly, this study did not consider the volume and expertise of the centres and surgeons involved, which have been shown to have substantial impact on mortality and morbidity rates in previous works^[18-22]^. Finally, this meta-analysis only included three studies. Hence, the analysis results may not be broadly representative of patients receiving thoracic aortic surgery, and the statistical representativeness of the study is inevitably limited due to the few studies included.

## CONCLUSION

The results from this meta-analysis demonstrate no significant difference in perioperative outcomes of using either axillary or IA cannulation during thoracic aortic surgery. Given the limitations of the analysis which includes only three comparative studies, the results must be interpreted carefully, and this highlight the need of a randomized trial comparing both techniques to understand the potential differences between each cannulation option on a larger, multi-centre level. Until then, the choice of site for cannulation in thoracic aortic surgery remains upon the operating surgeon's preference.

**Table:** 

Authors' roles & responsibilities	
AH	Substantial contributions to the conception or design of the work; or the acquisition, analysis, or interpretation of data for the work; drafting the work or revising it critically for important intellectual content; final approval of the version to be published
JSKC	Substantial contributions to the conception or design of the work; or the acquisition, analysis, or interpretation of data for the work; drafting the work or revising it critically for important intellectual content; final approval of the version to be published
CB	Substantial contributions to the conception or design of the work; or the acquisition, analysis, or interpretation of data for the work; drafting the work or revising it critically for important intellectual content; final approval of the version to be published
AH	Substantial contributions to the conception or design of the work; or the acquisition, analysis, or interpretation of data for the work; drafting the work or revising it critically for important intellectual content; final approval of the version to be published.
MS	Substantial contributions to the conception or design of the work; or the acquisition, analysis, or interpretation of data for the work; drafting the work or revising it critically for important intellectual content; final approval of the version to be published
SF	Substantial contributions to the conception or design of the work; or the acquisition, analysis, or interpretation of data for the work; drafting the work or revising it critically for important intellectual content; final approval of the version to be published
MB	Substantial contributions to the conception or design of the work; or the acquisition, analysis, or interpretation of data for the work; drafting the work or revising it critically for important intellectual content; final approval of the version to be published
